# Perceptual Constancy With a Novel Sensory Skill

**DOI:** 10.1037/xhp0000888

**Published:** 2020-12-03

**Authors:** Liam J. Norman, Lore Thaler

**Affiliations:** 1 Department of Psychology, Durham University

**Keywords:** constancy, blindness, echolocation, perceptual learning

## Abstract

Making sense of the world requires perceptual constancy—the stable perception of an object across changes in one’s sensation of it. To investigate whether constancy is intrinsic to perception, we tested whether humans can learn a form of constancy that is unique to a novel sensory skill (here, the perception of objects through click-based echolocation). Participants judged whether two echoes were different either because: (a) the clicks were different, or (b) the objects were different. For differences carried through spectral changes (but not level changes), blind expert echolocators spontaneously showed a high constancy ability (mean *d*′ = 1.91) compared to sighted and blind people new to echolocation (mean *d*′ = 0.69). Crucially, sighted controls improved rapidly in this ability through training, suggesting that constancy emerges in a domain with which the perceiver has no prior experience. This provides strong evidence that constancy is intrinsic to human perception.

## Public Significance Statement

This study shows that people who learn a new skill to sense their environment - here: listening to sound echoes - can correctly represent the physical properties of objects. This result has implications for effectively rehabilitating people with sensory loss.

Making sense of the world requires perceptual constancy—the stable perception of an object across changes in one’s sensation of it. A classic example of this is size constancy in vision, which can be described as the accurate judgment that an object has remained the same physical size despite viewing it from different distances (Holway & Boring, 1941). It is at present unclear to what degree perceptual constancy is intrinsic to human sensory processing. Supporting this idea, constancy can be found across all modalities (e.g., in vision, hearing, and touch; Fieandt, 1951; Sperandio & Chouinard, 2015; Yoshioka et al., 2011; Zahorik & Wightman, 2001) and in some limited forms can be present from birth (Slater et al., 1990) or from a very young age (7 months; Yang et al., 2015). Yet, the question of whether constancy is intrinsic to human sensory processing remains unanswered. Irrefutable evidence in support of this would require that people show constancy in an entirely novel sensory modality. Although it is not possible to demonstrate this, we can nonetheless test whether humans show constancy when using their existing senses to perceive objects in an entirely novel way: that is, using a new sensory skill. A new sensory skill allows a person to use a new sensory substitution or augmentation system. To this end, here we tested adults in their ability to achieve constancy using click-based echolocation—a sensory skill with which humans are typically unfamiliar, but which can be acquired through experience.

Echolocation is an acoustic method of sensing the world through sound reflections (Griffin, 1944). Human echolocators typically use mouth clicks to ensonify the world around them (Kolarik et al., 2014; Thaler & Goodale, 2016), and the returning echoes can be used to identify many physical properties of objects (e.g., size, shape, material; Milne et al., 2014, 2015; Teng & Whitney, 2011). Echolocation is mediated through hearing. It is a skill that most people are unfamiliar with, but which can be acquired through training (Dodsworth et al., 2020; Teng & Whitney, 2011; Tonelli et al., 2016). As such, click-based echolocation is an example of a novel sensory skill which allows a person to use a new sensory substitution or augmentation system (similar to devices like ‘The Voice,” a head-mounted device that translates visual scenes into acoustic signals; Meijer, 1992; and other sensory substitution devices; for a review, see Maidenbaum et al., 2014).

**Figure 1 fig1:**
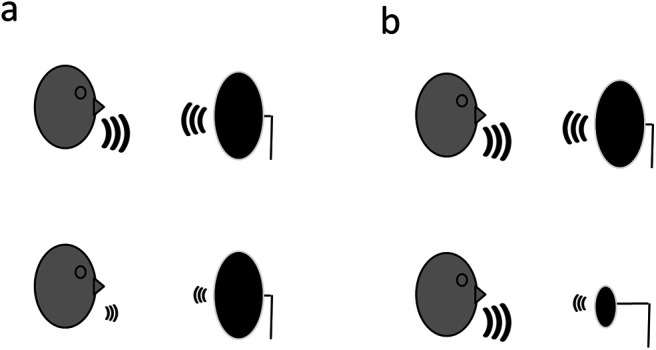
Two Scenarios (a and b) Are Depicted Here to Illustrate the Need for Constancy in Click-Based Echolocation *Note.* In Scenario a, the echolocator makes mouth clicks at different loudness levels—loud (top) and soft (bottom) at objects that are the same physical size. Due to the difference in the level of the clicks, there is also a relative difference in the level of the echoes reflected from the object—the echo from the top object is louder than that from the bottom object. Alternatively, in Scenario b, the echolocator makes identical clicks (top and bottom) but the object on the bottom is physically smaller than that on the top. As in Scenario a, this also results in a relative difference in the levels of the echoes. Therefore, in order to achieve constancy for the physical size of the object, an echolocator must resolve the ambiguity presented by these two scenarios. Variations in the spectrum of the echo can also vary for similar reasons—either because there is variation in the spectrum of the click or variation in the physical properties of the object (e.g., shape, material, size).

Studying constancy in human echolocation is ideally suited to testing whether humans show constancy in a novel sensory skill because not only is echolocation a sensory skill that most people have no experience with, but it also potentially holds forms of constancy that are entirely native and specific to the processes of echolocation. Constancy in click-based echolocation could be considered as the ability to perceptually represent the physical properties of the reflecting object (i.e., the distal stimulus) and not simply the raw sensory response elicited by the echo (i.e., the proximal stimulus). For expert echolocators (EEs), the level and spectrum of an echo carry information that can be used to recover the physical properties of the reflecting object such as its size, shape, and material (e.g., Milne et al., 2014, 2015; Teng & Whitney, 2011; Yu et al., 2018). This is possible because those properties of the reflecting object determine how much energy of the echolocator’s click is reflected at different wavelengths. The level and spectrum of the echo, however, are also determined by the level and spectrum of the echolocator’s click that is used to ensonify the object (Figure 1). For example, the level of the echo can increase either because (a) the echolocator increases the level of their click, or (b) the reflecting object increases in size. Similarly, the spectrum of the echo is also determined both by the initial spectrum of the click as well the various reflecting properties of the object (e.g., material, size, shape, etc.). Given that there is click-to-click variability in the level and spectrum of an EE’s click (de Vos & Hornikx, 2017; Thaler et al., 2017, 2018; Zhang et al., 2017), it follows that there are problems of perceptual constancy that must be solved by human echolocators.[Fig-anchor fig1]

These possible forms of constancy in echolocation are unlike those observed in other forms of novel sensory processing that function through the use of devices that translate information from one modality to another (e.g., see Maidenbaum et al., 2014). Visually impaired people can use such devices to recognize objects whose visual properties are translated into auditory information (Auvray et al., 2007), and can even show constancy by accurately perceiving size and orientation across variations in the angle at which the device captures the visual information (Stiles et al., 2015). With such examples of constancy, however, the relevant sensory relationships that must be disambiguated to achieve constancy are not native to the novel sensory skill—they are defined with respect to their original modality and are translated from their original modality into the modality used for substitution (e.g., from vision to audition in the case of Stiles et al., 2015). Although the stimulus coding space might be entirely different across that translation (e.g., converting a spatial dimension into one of frequency), it remains possible that constancy is solved only through cross-modal imagery in the stimulus’ original coding space (e.g., see Spence & Deroy, 2013, for a discussion of cross-modal mental imagery). It follows that by testing whether people show a form of constancy that is native and specific to echolocation, we can provide the most direct and unambiguous evidence that constancy can be learned by humans using a novel sensory skill.

Here, we define constancy in echolocation as the ability to correctly attribute a change in the echo to a change either in the emission or the reflecting object. This is a performance-based “operational” approach to measuring constancy, which has its origins in studies of color constancy (Craven & Foster, 1992). We chose this approach for two reasons: (a) it does not rely on the subject being able to identify or perceptually match the properties of the reflecting object, and (b) it is a form of constancy that is achieved with high accuracy and little cognitive effort by subjects when compared to alternative measures (Craven & Foster, 1992; Foster et al., 1992). In our constancy tasks, participants listened to two click-echo pairs and judged whether the difference between echoes across the two pairs (either in level or spectrum, separately) was a result of variation in the clicks’ acoustic properties or in the objects’ reflecting properties.

In Experiments 1–3 we tested people’s ability to show constancy across variations in the echo’s spectrum. In Experiments 4 and 5 we tested people’s ability to show constancy across variations in the echo’s level. We also considered the effect of echolocation experience in this context by testing expert echolocators (EEs) as well as blindfolded sighted controls (SCs) and blind controls (BCs) with no prior experience in echolocation. We include both BCs and SCs in order to determine whether any superior ability of EEs is due to visual impairment alone. Given the previous work showing that both spectral composition and sound level are important perceptual features in click-based echolocation in humans (e.g., Norman & Thaler, 2020), and that EEs perform better than both BCs and SCs in tasks that involve passively listening to echolocation sounds (e.g., Norman & Thaler, 2020), we predict that EEs will show constancy across variations in the echo’s spectrum and level, and they will perform better than both BCs and SCs in this ability. We do not expect BCs and SCs to differ in their ability. If the superior constancy in EEs is driven by expertise in echolocation, then SCs should improve in this ability with training for both spectrum and level (Experiments 3 and 5, respectively).

## General Materials and Methods

All experiments reported in this study share some common elements, which are described below.

### Ethics

All procedures followed the British Psychological Society code of practice and the World Medical Association’s Declaration of Helsinki. The experiment had received ethical approval by the Ethics Advisory Sub-Committee in the Department of Psychology at Durham University. All participants gave written informed consent to take part in this study.

### Participants

Three participant groups were tested—EEs, BCs, and SCs. BCs and SCs reported having no prior experience with click-based echolocation, except for two of the BCs, who had taken part in a previous study in our lab which had required them to listen to echolocation sounds and to make clicks, but who did not meet our criteria for EEs in terms of regularity and duration of use of echolocation. In Experiments 3 and 5 (the training experiments), only sighted participants were tested. Those who were classed as EEs reported using click-based echolocation on a daily basis for more than 10 years. Participants had normal hearing, with the exceptions of BC7, BC10, and BC17 who had some loss for frequencies beyond 4 kHz consistent with their age. Table 1 shows relevant details of the EEs and BCs who took part (i.e., age, gender, degree and cause of vision loss, age at onset of vision loss). Some participants took part in more than one of the experiments reported here and Table 1 shows which experiment each participant took part in. Participants were compensated either at a rate of £10/hr or with course credit. SCs were recruited through internal advertising within the Durham University Department of Psychology. BCs were recruited through word-of-mouth. All EEs had taken part in studies with us before, and were recruited for this study through direct invitations.[Table-anchor tbl1]

### Statistical Power

We had practical limitations on our sample sizes for EEs and BCs. In order to demonstrate that we have sufficient power and precision to support our statistical inferences, we calculated the minimum effect size that can be detected with our sample sizes. We did this separately for the four types of critical statistical tests that we use to support our main conclusions. These tests are: (a) testing whether each participant group performs better than chance in the constancy tasks (Experiments 1, 2, and 4), (b) testing whether there is a difference between groups’ performance in the constancy tasks (Experiments 1 and 4), (c) testing whether constancy performance across variations in spectrum is affected by the intensity of the echoes (Experiment 2), and (d) testing whether performance in the constancy tasks improves with training (Experiments 3 and 5). For all of these tests, we used G*Power 3.1.9.7 (Faul et al., 2007) to compute required effect sizes (for two-tailed tests), setting α to 0.05 and power to 0.8. Where G*Power computes effect sizes as Cohen’s *f*, these values were converted to η^2^ or η_*p*_^2^ values to be consistent with the units of our reported effect sizes. These computed minimum effect sizes are reported throughout this article alongside the observed effect sizes for any critical tests that are statistically significant, with additional details provided for each test where necessary.

### Apparatus and Recording Process

#### Recording Process

**Figure 2 fig2:**
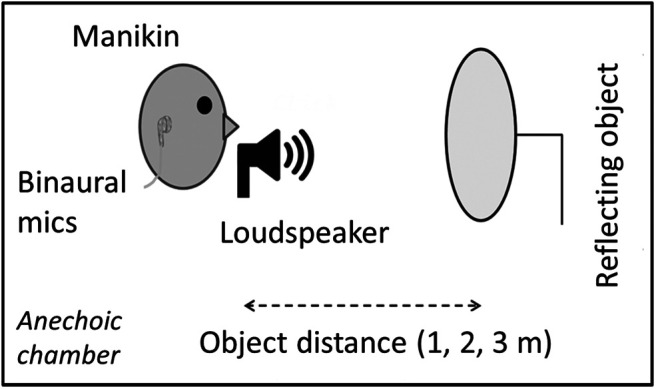
Sketch of the Apparatus Setup Used for Making the Sound Recordings *Note.* A manikin was positioned behind a loudspeaker, which emitted a click. A wooden disk was used as a reflecting object and positioned at a distance of either 1, 2, or 3 m from the loudspeaker, or not present at all. Recordings were made using binaural microphones.

The stimuli for these experiments were created from recordings of echolocation sounds that we made for a previous set of experiments (Norman & Thaler, 2020). The recording process is described in detail in that publication, but some important details are summarized below. The setup of the recording apparatus is shown in Figure 2.[Fig-anchor fig2]

#### Recording Clicks With Varying Spectra

Three variations in the click’s peak spectrum were used: 3.5, 4.0, and 4.5 kHz—hereafter referred to as low, medium, and high frequencies, respectively—and across these variations the level of the emissions was held constant. We chose these peak frequencies as they reflect a range that is found in natural human mouth clicks of EEs (Thaler et al., 2017; Zhang et al., 2017). It should be noted that emissions containing higher spectral frequencies lead to stronger echoes being reflected from the target object because, for an object of fixed proportions, sound composed of shorter wavelengths will be more strongly reflected than one composed of longer wavelengths. Thus, the echoes are more intense as the peak spectrum of the emission is increased. These natural variations are preserved in Experiment 1, and in Experiment 2 we directly assess whether the presence of these level differences is necessary for constancy.

#### Recording Clicks With Varying Levels

Three variations in the click’s level were acquired by digitally amplifying the emission sound by factors of 0 dB (i.e., baseline), −3 dB, and −6 dB (using the “Amplify” function in Audacity(R) 2.1.2; Audacity Team, 2016) – hereafter referred to as high, medium and low levels, respectively. The peak spectrum was held constant at 4.5 kHz.

#### Creating the Stimuli for the Constancy Task

In preparing the sounds to be used as stimuli in the constancy task, and also in one of the training tasks described below, it was first necessary to be able to digitally separate the click and echoes at each target distance level. This was needed in order to be able to digitally recombine clicks and echoes from recordings with different emission levels or spectral frequencies—for example, to create a high level click with a low level echo—which allows us to simulate the presence of an object of varying reflecting properties. While this virtual approach might lead to click-echo combinations that are unlikely to arise in everyday situations, it gives us precise control over the acoustic properties of clicks and echoes. The temporal onset of the echo at each target distance was identified by visual inspection of the waveforms, with the point at which the waveform first rose above the noise floor being taken as the temporal onset of the echo. Any sound data recorded after this point were taken as belonging to the echo, and any before this point were taken as belonging to the click emission.

#### Behavioral Experiments

Participants were tested in the same sound-insulated and echo-acoustic dampened room in which the sound recordings had been made (described in Norman & Thaler, 2020). Sounds were played to participants through binaural in-ear headphones (Etymotic Research ER4B MicroPro; ETYMOTIC RESEARCH, INC., Elk Grove Village, Illinois) driven by a Dell Latitude E7470 laptop (Intel Core i56300U CPU 2.40 GHz, 8 GB RAM, 64-bit Windows 7 Enterprise) through a USB soundcard (Creative Sound Blaster X-Fi HD sound card; Creative Technology, Creative Labs Ireland, Dublin, Ireland). Sounds were played to participants at a level at which the sound file with the highest peak level was presented at 80-dB sound pressure level. Participants sat upright and gave their response using a keyboard. Participants who were not fully blind wore a blindfold.

For participants to successfully show perceptual constancy for an object across variations in the echo, they must first be able to recognize when an echo is present (compared to when it is absent). They must also be able to discriminate the variations in the acoustic properties of the echo and emission that are relevant to the constancy task. Thus, participants completed three echo-acoustic training tasks prior to completing the constancy task. In each of these tasks they either (a) detected the presence of an echo, (b) discriminated differences in the echo’s spectrum or level, or (c) discriminated differences in the emission’s spectrum or level (with no echo present).

In each task, participants pressed a key to begin each trial. Each task consisted of a two-alternative forced choice task, where two sounds were played to participants consecutively with an inter stimulus interval of 1 s. The two sounds on each trial were played in a random order determined on each trial and participants then pressed one of two keys on a keyboard to indicate their response. During the three echo-acoustic training tasks, but not during the constancy task itself (except in Experiments 3 and 5), participants received auditory feedback (2500-Hz “correct” tone or 600-Hz “incorrect” 50-ms tone) on each trial to indicate whether they were correct or not. Before each task, participants were given a practice block that was one third the length of the main block.

#### Echo-Acoustic Training: Echo Detection

Participants judged whether an echo was present in the first or second of two sounds. On each trial, emissions of the same spectrum/level were used (both either high or low^1^). After one of these emissions the echo from an object at either 1, 2, or 3 m was present, and after the other no echo was present. For each emission level (low/high), each of these target distances was tested 15 times, amounting to a total of 90 trials per block. Proportion correct was then calculated and averaged across levels of target distance for each participant.

#### Echo-Acoustic Training: Echo Discrimination

Participants judged which of two echoes was higher in pitch or level. On each trial, emissions of the same spectrum/level were used (medium) and, after both of these emissions, the echo from an object at a distance of either 1, 2, or 3 m was present. One of these echoes was taken from the low spectrum/level emission recording, and the other taken from the high spectrum/level emission recording. (see Footnote 1) Each target distance was tested 15 times in each block, amounting to a total of 45 trials per block. Proportion correct was then calculated and averaged across levels of target distance for each participant.

#### Echo-Acoustic Training: Emission Discrimination

Participants judged which of two click emissions was either higher in pitch or level. The low and high spectrum/level emissions were played in a random order on each trial, and there was no echo present in either sound. This was repeated 15 times, amounting to a total of 15 trials per block. Proportion correct was then calculated for each participant.

#### Constancy Task

**Figure 3 fig3:**
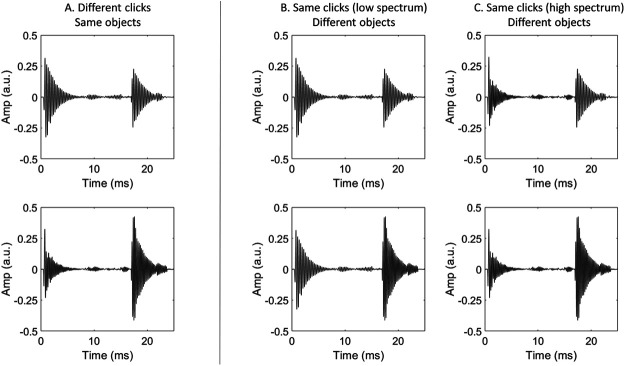
Waveforms of the Stimuli Used to Measure Constancy Across Variations in Spectrum *Note.* In each trial, subjects heard two sound recordings—both containing a click and echo from a target object. The echoes were always different in their spectrum. On 50% of trials, this spectrum difference was due to the clicks also being different in their spectrum (Column A). On the remaining 50% of trials, the clicks were either the same low spectrum (Column B; 25%) or same high spectrum (Column C; 25%). In these latter two cases, the relative difference in spectrum of the echoes can only be explained by differences in the reflecting properties of the object. Subjects’ task was to judge whether the echoes were different either because the clicks were different or because the reflecting objects were different. Only the echoes from a 3-m target are shown here—echoes from 1- and 2-m targets were also used in the experiment. The *y*-axis shows amplitude in arbitrary units (a.u.).

**Figure 4 fig4:**
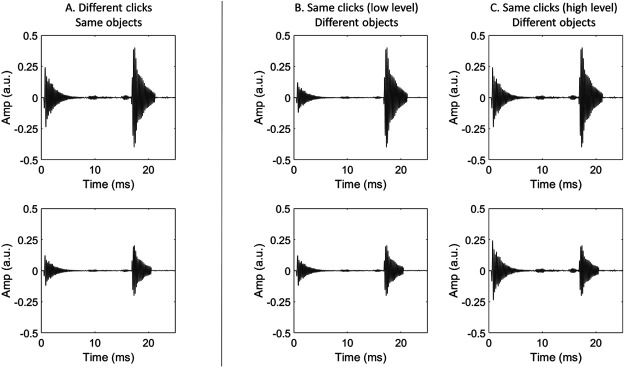
Waveforms of the Stimuli Used to Measure Constancy Across Variations in Level *Note.* The design was the same as that described for variations in spectrum (see Figure 3), but here the clicks and echoes vary in level and not spectrum. The *y*-axis shows amplitude in arbitrary units (a.u.).

Participants judged whether two echoes were different in their spectrum (Experiments 1–3) or level (Experiments 4–5) either because (a) the clicks were different, or (b) the objects were different. On each trial the echoes were always different in their spectrum/level (high/low), and were reflected from an object at the same distance (either 1, 2, or 3 m). On half of the trials, the emissions were different to one another in their spectrum/level (high/low), and these matched the spectrum/level of their respective echoes (i.e., a low level click, followed by a low level echo). In the remaining half of the trials, the two emissions had the same spectrum/level (either low or high, occurring equally often). Thus, in trials in which the echoes varied with the clicks, the correct response was to judge that the echoes were different because the clicks were different. Alternatively, in trials in which the echoes did not vary with the clicks, the correct response was to judge that the echoes were different because the objects were different. Figures 3 and 4 display examples of the stimuli used in the spectrum and level constancy tasks, respectively. There were 60 trials for each target distance in each block, amounting to a total of 180 trials in each block. Before each constancy task, participants were told explicitly whether the differences would be carried by differences in level or spectrum.[Fig-anchor fig3][Fig-anchor fig4]

Unlike the echo-acoustic training tasks, which were two-interval forced choice tasks, the constancy task required participants to classify each trial in one of two ways (i.e., “objects different” or “clicks different”). Thus, it is possible that response bias affected participant’s performance in the constancy task, and therefore a bias-free measure of performance (*d*′) was calculated from hit rates and false alarm rates [*d*′ = *z*(HR) – *z*(FAR)]. Hits were classed as trials in which the participant correctly identified that the echoes were different because the objects were different. False alarms were classed as trials in which participants judged that the echoes were different because the objects were different, when in fact the clicks were different. A higher *d*′ indicates a greater ability to accurately classify the two types of trial (i.e., greater constancy ability), and a *d*′ of zero indicates no ability to do this (i.e., no constancy).

## Experiment 1: Constancy Across Variations in Spectrum

In Experiment 1 we tested people’s ability to show constancy across variations in the echo’s spectrum

### Participants

Three EEs (3 males; *M*_age_ = 36.33; *SD* = 13.05), 10 BCs (7 males; *M*_age_ = 52.80; *SD* = 11.96) and 10 SCs (2 males; *M*_age_ = 22.40; *SD* = 2.72) took part in Experiment 1.

### Results

#### Echo-Acoustic Training: Echo Detection

**Figure 5 fig5:**
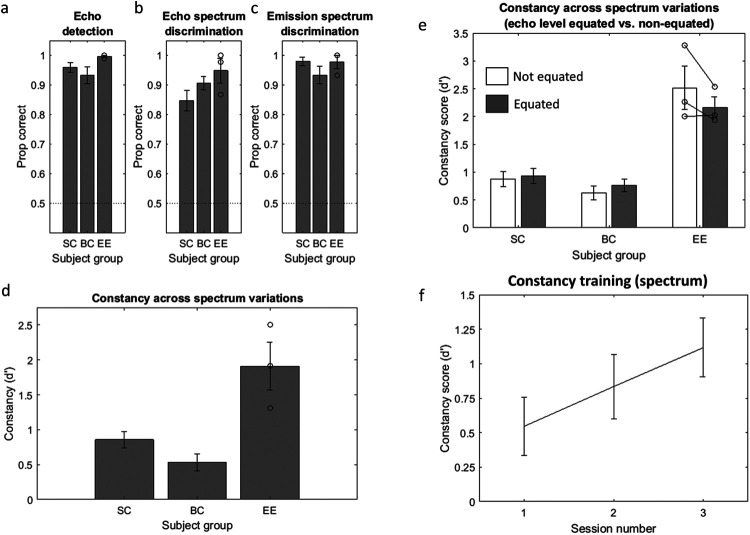
Results From Experiments 1 (a-d), 2 (d) and 3(f) *Note.* In Experiment 1, subjects completed 3 echo-acoustic training tasks (a–c) before completing the constancy task (d). In Experiment 2, (e), subject’s constancy ability across variations in spectrum arose regardless of differences in the level of the echoes. In Experiment 3, sighted subjects new to echolocation were trained on the constancy task over a period of three separate days. Their performance on the spectrum task improved rapidly with training.

On average, each subject group performed very well (Figure 5a), and, as shown with one-sample *t* tests, each group performed significantly better than chance (SC: *M* = .96, *t*(9) = 28.88, *p* < .001, *d* = 9.13; BC: *M* = .93, *t*(9) = 15.06, *p* < .001, *d* = 4.76; EE: *M* = 1.00, *t*(2) = 134.01, *p* < .001, *d* = 77.36). An independent analysis of variance (ANOVA) with group as between-subjects factor showed there was no difference between groups, *F*(2, 22) = 1.06, *p* = .37, η^2^ = .10. Using modified *t* tests for testing whether a single case differs significantly from a control group (Crawford & Garthwaite, 2002; Crawford & Howell, 1998), compared to the combined SC and BC participants (*M* = .95, *SD* = 0.07), none of the EEs performed significantly differently (EE1 = .99, *t*_(19)_ = 0.58, *p* = .57; EE2 = 1.00, *t*_(19)_ = 0.73, *p* = .47; EE3 = 1.00, *t*_(19)_ = 0.73, *p* = .47).[Fig-anchor fig5]

#### Echo-Acoustic Training: Echo Spectrum Discrimination

On average, each subject group performed very well (see Figure 5b), and, as shown with one-sample *t* tests, each group performed significantly better than chance (SC: *M* = .85, *t*(9) = 10.14, *p* < .001, *d* = 3.21; BC: *M* = .91, *t*(9) = 18.14, *p* < .001, *d* = 5.74; EE: *M* = .95, *t*(2) = 10.87, *p* = .008, *d* = 6.27). An independent ANOVA with group as between-subjects factor showed there was no difference between groups, *F*(2, 22) = 1.95, *p* = .17, η^2^ = .16. Using modified *t* tests, compared to the combined SC and BC participants (*M* = .88, *SD* = 0.09), none of the EEs performed significantly differently (EE1 = .98, *t*_(19)_ = 1.05, *p* = .31; EE2 = 1.00, *t*_(19)_ = 1.28, *p* = .22; EE3 = .87; *t*_(19)_ = 0.10, *p* = .92).

#### Echo-Acoustic Training: Emission Spectrum Discrimination

On average, each subject group performed very well (see Figure 5c), and, as shown with one-sample *t* tests, each subject group performed significantly better than chance (SC: *M* = .98, *t*(9) = 33.74, *p* < .001, *d* = 10.67; BC: *M* = .95, *t*(9) = 15.15, *p* < .001, *d* = 15.20; EE: *M* = .98, *t*(2) = 21.49, *p* = .002, *d* = 12.41). An independent ANOVA with group as between-subjects factor showed there was no difference between groups, *F*(2, 22) = 1.19, *p* = .33, η^2^ = .11. Using modified *t* tests, compared to the combined SC and BC participants (*M* = .88, *SD* = 0.08), none of the EEs performed significantly differently (EE1 = 1.00, *t*_(19)_ = 0.67, *p* = .51; EE2 = 1.00, *t*_(19)_ = 0.67, *p* = .51; EE3 = .93, *t*_(19)_ = 0.31, *p* = .76).

#### Constancy Task

Unlike the training tasks, which measured performance using proportion correct, the constancy task measured performance using *d*′, where a score of zero indicates no ability to discriminate (i.e., chance). On average, each subject group performed well (see Figure 5d), and, as shown with one-sample *t* tests, performed significantly better than chance (SC: *M* = 0.80, *t*(9) = 8.79, *p* < .001, *d* = 2.96; BC: *M* = .55, *t*(9) = 5.77, *p* < .001, *d* = 1.76; EE: *M* = 1.63, *t*(2) = 6.61, *p* = .025, *d* = 4.16). The minimum detectable effect size was calculated to be *d* = 1.00 for SCs and BCs, and *d* = 3.26 for EEs. Thus, even people who had no prior experience in click-based echolocation (i.e., SCs and BCs) demonstrated constancy. Furthermore, however, an independent ANOVA with group as between-subjects factor showed there was a difference between groups, *F*(2, 22) = 13.62, *p* < .001, η^2^ = .57, with EEs performing better and having a higher *d*′ (*M* = 1.63) compared to sighted (*M* = 0.80; *p* = .002) and blind (*M* = 0.55; *p* < .001) controls. The minimum detectable effect size^2^ was computed to be η^2^ = 0.33. There was no significant difference between BCs and SCs (*p* = .26). Using modified *t* tests, compared to the combined SC and BC participants (*M* = 0.63, *SD* = 0.40), two of the EEs performed significantly better (EE1 = 2.50, *t*_(19)_ = 4.37, *p* < .001; EE2 = 1.92, *t*_(19)_ = 2.96, *p* = .008) and one did not (EE3 = 1.31, *t*_(19)_ = 1.50, *p* = .15).

A separate analysis was run to test whether performance on this task varied with target distance. A repeated measures ANOVA with the within-subject factor target distance (1, 2, 3 m) and the between-subjects factor subject group (EE, BC, SC) revealed a significant effect of target distance, *F*(2, 40) = 7.19, *p* = .002, η_*p*_^2^ = .26 with a significant negative linear trend, *F*(1, 20) = 16.16, *p* = .001, η^2^ = .45. Mean *d*′ decreased from 1.00 (1 m) to 0.85 (2 m) and 0.71 (3 m), that is, people performed better with echoes from objects at closer distances. There was no interaction between target distance and subject group, *F*(4, 40) = 1.51, *p* = .22, η_*p*_^2^ = .13.

## Experiment 2: Constancy Across Variations in Spectrum (With Echo Level Equated)

Experiment 2 was designed to address whether the results in Experiment 1 can be attributed to the associated naturally occurring variations in level of the echo. Specifically, although the level of the click emission was matched in Experiment 1, the level of the echo varied with the spectrum of the click emission (this can be seen in the images in Figure 3). This effect is expected because sound waves of higher frequencies are composed of shorter wavelengths, which lead to stronger echoes from an object of the size used here. In Experiment 2, therefore, we tested people’s ability to show constancy across variations in the echo’s spectrum, while equating the level of the echo across variations in its spectrum.

### Method

#### Participants

3 EEs (3 males; *M*_age_ = 36.33; *SD* = 13.05), 10 BCs (9 males; *M*_age_ = 48.00; *SD* = 12.36) and 10 SCs (4 males; *M*_age_ = 23.60; *SD* = 3.20) took part in Experiment 2.

#### Sound Processing

Sound recordings that were made across variations in the emission’s spectrum (see the Recording Clicks With Varying Spectra section) were digitally processed in order to equate the level of the echo across these variations. In order to equate the level of the echoes across variations in spectrum, the temporal onset of the echo at each level of target distance first had to be identified. This was done by visual inspection of the waveforms for the 4.0-kHz click recordings, with the point at which the waveform first rose above the noise floor being taken as the temporal onset of the echo. Any sound data following these identified time points in the recordings of the 3.5-kHz and 4.5-kHz emissions were then multiplied by respective scaling factors in order to equate their peak level to that in the recording of the 4.0-kHz emission. Sounds for the constancy task were then digitally edited in the same way as previously described (see the Creating the Stimuli for the Constancy Task section*).*

#### Procedure

We used the sound recordings from Experiment 1 to create a further set of sounds in which the peak level of the echo had been equated across levels of the emission’s spectrum (separately for each level of target distance). The constancy task was then run using these new sounds. Each participant completed two blocks of the original constancy task (echo level not equated) and two blocks of the modified constancy task (echo level equated). The order of these blocks was counterbalanced across participants. *d*′ was calculated separately for the two versions of the constancy task (level equated, level not equated) and for each target distance.

Participants did not take part in the echo-acoustic training tasks as part of this experiment, but some participants (3 EEs; 6 BCs) had also completed them as part of Experiment 1 (for details see Table 1). Importantly, Experiment 2 is concerned with within-subject differences in performance between equated and nonequated sound conditions, so that performance in echo-acoustic training tasks is of no relevance.

### Results

Figure 5e shows the results (collapsed across distance levels). Just as in Experiment 1 each subject group performed well (see Figure 5e), and, as shown with one-sample *t* tests, performed significantly better than chance both in nonequated (SC: *M* = 0.88, *t*(9) = 6.56, *p* < .001, *d* = 2.07; BC: *M* = 0.63, *t*(9) = 5.23, *p* = .001, *d* = 1.65; EE: *M* = 2.52, *t*(2) = 6.44, *p* = .023, *d* = 3.72) and equated conditions (SC: *M* = 0.93, *t*(9) = 6.71, *p* < .001, *d* = 2.12; BC: *M* = 0.77, *t*(9) = 6.71, *p* < .001, *d* = 2.12; EE: *M* = 2.17, *t*(2) = 11.54, *p* = .007, *d* = 6.66). The minimum detectable effect size was calculated to be *d* = 1.00 for SCs and BCs, and *d* = 3.26 for EEs. Thus, again, even people who had no prior experience in click-based echolocation (i.e., SCs and BCs) demonstrated constancy.

A mixed-model ANOVA was carried out, with the within-subject factors echo level (equated, not equated) and target distance (1 m, 2 m, 3 m), and the between-subjects factor participant group (EE, BC, SC). As to the main objective of Experiment 2—to address if constancy found in Experiment 1 could be due to variations in level of the echo—there was no significant effect of echo level, *F*(1, 20) = 0.37, *p* = .55, η_*p*_^2^ = .02. Thus, constancy due to spectral variations is not affected by level. This is also shown by the data in Figure 5e. The three-way interaction was significant, *F*(4, 40) = 6.67, *p* < .001, η_*p*_^2^ = .40. In order to further explore this, we followed up with two-way ANOVAs, separately for equated and nonequated conditions.

For nonequated conditions, the analysis largely replicated the findings from Experiment 1—a significant main effect of group, *F*(2, 20) = 22.15, *p* < .001, η_*p*_^2^ = .69, with EEs (*M* = 2.51) performing better than SCs (*M* = 0.88; *p* < .001) and BCs (*M* = 0.63; *p* < .001), and a significant effect of distance, *F*(2, 40) = 5.12, *p* = .010, η_*p*_^2^ = .20. The interaction between subject group and distance was also significant, *F*(4, 40) = 5.37, *p* = .001, η_*p*_^2^ = .35, and follow-up *t* tests revealed that this was due to the fact that EEs were significantly better than both SCs and BCs at 1 m, *t*(11) = 5.78, *p* < .001, *d* = 4.14; *t*(11 = 12.03, *p* < .001, *d* = 6.80) and 2 m, *t*(11) = 4.94, *p* < .001, *d* = 2.16; *t*(11 = 5.12, *p* < .001, *d* = 2.40), but not 3 m, *t*(11) = 1.78, *p* = .10, *d* = 1.03; *t*(11 = 2.14, *p* = .06, *d* = 1.21). For equated conditions, the analysis again revealed a main effect of group, *F*(2, 20) = 14.93, *p* < .001, η_*p*_^2^ = .60, with EEs (*M* = 2.17) performing better than SCs (*M* = 0.93; *p* < .001) and BCs (*M* = 0.77; *p* < .001), but the effect of distance, *F*(2, 40) = 2.14, *p* = .13, η_*p*_^2^ = .10 and the Distance × Group interaction, *F*(4, 40) = 2.19,*p* = .09, η_*p*_^2^ = .18 were not significant.

## Experiment 3: Learning Constancy in Echolocation (Spectrum)

In Experiment 1 and 2, while all subject groups had demonstrated constancy, EEs also showed better constancy performance relative to control subjects. The purpose of Experiment 3 was to determine whether SCs could significantly improve in their constancy ability across three short constancy training sessions.

### Method

#### Participants

Ten SCs (3 males; *M*_age_ = 22.80; *SD* = 2.97) took part in Experiment 3.

#### Procedure

Each participant completed four separate sessions of testing, and each session was conducted at least 24 hr after the last. In the first session, participants completed the three echo-acoustic training tasks for the spectrum conditions. In each of the following three sessions they completed two blocks of the spectrum constancy task (with sounds in which the echoes were not equated for level, as in Experiment 1). Each session lasted only 30 min and auditory feedback was given to participants after each trial to indicate whether they were correct or not.

### Results

Figure 5f shows the results. A one-way repeated measures ANOVA showed a significant effect of session number on *d*′, *F*(2, 18) = 4.29, *p* = .03, η_*p*_^2^ = .32. The minimum detectable effect size^3^ was computed to be η_*p*_^2^ = .23. There was a significant positive linear trend, *F*(1, 9) = 8.10, *p* = .019, η_*p*_^2^ = .47, with *d*′ increasing^4^ from 0.55 in Session 1, to 0.83 in Session 2 and 1.12 in Session 3. This is consistent with the idea that the difference in performance that we observed between EEs and controls was due to the experts’ experience, and that this can be rapidly acquired also by sighted people new to the skill.

## Experiment 4: Constancy Across Variations in Level

The general procedure was the same as that used in Experiment 1, except using sounds with variations in level not spectrum.

### Participants

3 EEs (3 males; *M*_age_ = 36.33; *SD* = 13.05), 10 BCs (7 males; *M*_age_ = 57.00; *SD* = 12.54) and 10 SCs (3 males; *M*_age_ = 23.20; *SD* = 3.97) took part in Experiment 4.

### Results

#### Echo-Acoustic Training: Echo Detection

**Figure 6 fig6:**
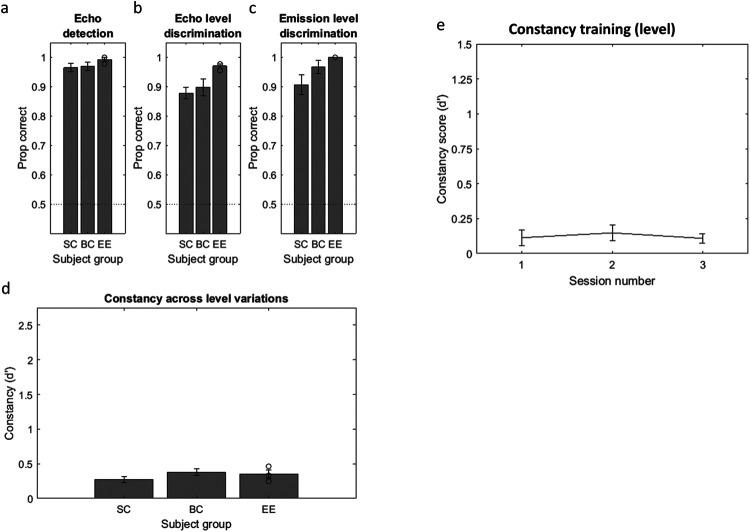
Results From Experiments 4 (a–d) and 5 (e) *Note.* In Experiment 4, subjects completed three echo-acoustic training tasks (a–c) before completing the constancy task (d). In Experiment 5, sighted subjects new to echolocation were trained on the constancy task over a period of three separate days. Their performance on the level task did not improve with training.

On average, each subject group performed very well (Figure 6a), and, as shown with one-sample *t* tests, each subject group performed significantly better than chance (SC: *M* = .96, *t*(9) = 33.09, *p* < .001, *d* = 10.46; BC: *M* = .97, *t*(9) = 32.31, *p* < .001, *d* = 10.21; EE: *M* = .99, *t*(2) = 66.57,*p* < .001, *d* = 38.39). An independent ANOVA with group as between-subjects factor showed there was no difference between groups, *F*(2, 22) = 0.50, *p* = .61, η^2^ = .05. Using modified *t* tests, compared to the combined SC and BC participants (*M* = .97, *SD* = 0.04), none of the EEs performed significantly differently (EE1 = .98, *t*_(19)_ = 0.25, *p* = .81; EE2 = 1.00, *t*_(19)_ = 0.74, *p* = .47; EE3 = 1.00; *t*_(19)_ = 0.74, *p* = .47).[Fig-anchor fig6]

#### Echo-Acoustic Training: Echo Level Discrimination

On average, each subject group performed very well (see Figure 6b), and, as shown with one-sample *t* tests, each subject group performed significantly better than chance (SC: *M* = .88, *t*(9) = 19.49, *p* < .001, *d* = 6.16; BC: *M* = .90, *t*(9) = 13.49, *p* < .001, *d* = 4.23; EE: *M* = .97, *t*(2) = 63.57, *p* < .001, *d* = 36.66). An independent ANOVA with group as between-subjects factor showed there was no difference between groups, *F*(2, 22) = 1.76, *p* = .20, η^2^ = .15. Using modified *t* tests, compared to the combined SC and BC participants (*M* = .89, *SD* = 0.08), none of the EEs performed significantly differently (EE1 = .98, *t*_(19)_ = 1.13, *p* = .27; EE2 = .96, *t*_(19)_= 0.85, *p* = .40; EE3 = .98; *t*_(19)_ = 1.13, *p* = .27).

#### Echo-Acoustic Training: Emission Level Discrimination

On average, each subject group performed very well (see Figure 6c), and, as shown with one-sample *t* tests, each subject group performed significantly better than chance (SC: *M* = .91, *t*(9) = 12.23, *p* < .001, *d* = 3.87; BC: *M* = .97, *t*(9) = 20.50, *p* < .001, *d* = 20.37; EE: *M* = 1.00, *t*(2) = inf, *p* < .001, *d* = inf). An independent ANOVA with group as between-subjects factor showed there was no difference between groups, *F*(2, 22) = 1.95, *p* = .17, η^2^ = .17. Using modified *t* tests, compared to the combined SC and BC participants (*M* = .94, *SD* = 0.09), none of the EEs performed significantly differently (EE1 = 1.00, *t*_(19)_ = 0.67, *p* = .51; EE2 = 1.00, *t*_(19)_ = 0.67, *p* = .51; EE3 = 1.00, *t*_(19)_ = 0.67, *p* = .51).

#### Constancy Task

On average, each subject group showed poor constancy (see Figure 6d), but, as shown with one-sample *t* tests, nonetheless performed significantly better than chance (SC: *M* = 0.27, *t*(9) = 6.15, *p* < .001, *d* = 1.94; BC: *M* = 0.38, *t*(9) = 7.99, *p* < .001, *d* = 2.53; EE: *M* = 0.35, *t*(2) = 5.53 *p* = .031, *d* = 3.20). The minimum detectable effect size was calculated to be *d* = 1.00 for SCs and BCs, and *d* = 3.26 for EEs. Although the observed effect size for the EE group is marginally lower than the minimum that is detectable, it is nonetheless a very large effect size and the number of participants who meet the selection criteria for this group is extremely small. An independent ANOVA with group as between-subjects factor showed there was no difference between groups, *F*(2, 22) = 1.46, *p* = .26, η^2^ = .13. Compared to the control participants (*M* = 0.33, *SD* = 0.15), none of the EEs performed significantly differently (EE1 = 0.25, *t*_(19)_ = 0.50, *p* = .62; EE2 = 0.47, *t*_(19)_ = 0.91, *p* = .38; EE3 = 0.34, *t*_(19)_ = 0.07, *p* = .94).

A separate analysis was run to test whether performance on this task varied with target distance. A repeated measures ANOVA with the within-subject factor target distance (1, 2, 3 m) and the between-subjects factor subject group (EE, BC, SC) revealed a significant effect of target distance, *F*(2, 40) = 3.93, *p* = .028, η_*p*_^2^ = .16 with a significant positive linear trend, *F*(1, 20) = 5.84, *p* = .025, η_*p*_^2^ = .23. Mean *d*′ increased from 0.20 (1 m) to 0.30 (2 m) and 0.48 (3 m), that is, people performed better with echoes from objects at farther distances. There was no interaction between target distance and subject group, *F*(4, 40) = 0.82, *p* = .52, η_*p*_^2^ = .08. As stated above, there was no difference across groups, *F*(2, 22) = 1.46, *p* = .26, η_*p*_^2^ = .13.

## Experiment 5: Learning Constancy in Echolocation (Level)

The general procedure was the same as that used in Experiment 3, except using sounds with variations in level not spectrum.

### Participants

Participants were those who took part in Experiment 3.

### Results

Figure 6e shows the results. A one-way repeated measures ANOVA showed no significant effect of session number on *d*′, *F*(2, 18) = 0.27, *p* = .77, η_*p*_^2^ = .03. These results suggest that constancy across variations in level does not improve with training, unlike that across variations in spectrum (Experiment 3).

## General Discussion

We found clear evidence that when people perceive objects through click-based echolocation, they show a type of perceptual constancy that is entirely native and specific to echolocation. Specifically, people were able to discriminate between changes in an object’s reflected sound that arose from (a) changes in the echolocator’s click, or (b) changes in the object’s reflective properties. Importantly, because this type of constancy is specific to click-based echolocation, it cannot be derived from another modality. Our results are therefore strong evidence that perceptual constancy is an intrinsic part of human sensory processing. Furthermore, our echo-acoustic training tasks confirmed that all participants could perceive the acoustic features relevant for the constancy task. Thus, performance in the constancy task is not limited by people’s ability/inability to perceive the relevant constituent sounds.

Although subjects were generally able to do the constancy tasks in the present study, there were some important differences in ability between conditions and subject groups. Constancy across variations in the level of the echo (Experiment 4) was comparably poor in all subject groups, and EEs were no better compared to the control groups. Conversely, constancy across variations in the spectrum of the echo (Experiments 1 and 2) was better overall, and EEs showed a significantly greater ability compared to controls. The clear advantage that EEs showed in the spectrum task relative to the level task suggests a functional independence between the processes underlying constancy across variations in level and spectrum. This advantage also reflects the results of previous studies that have shown the importance of spectral cues relative to level cues in tasks of echo detection and discrimination (Norman & Thaler, 2020; Schenkman & Nilsson, 2011). Furthermore, results from Experiment 3 suggest that constancy in the spectrum task was due to an expertise that could be rapidly acquired through learning, even in SCs. Thus, while we found that constancy is present even in a novel sensory skill, thus providing strong support for the idea that perceptual constancy is intrinsic to human sensory processing, we also found that the processes serving this skill with respect to spectral constancy in echolocation are affected by learning. Furthermore, this appears to be a fast learning process—constancy ability across variations in spectrum doubled on average over a period of three short (30 min) training sessions.

In many human models of perceptual constancy, relations between sensory channels (e.g., the ratios of cone activations in color constancy; Foster & Nascimento, 1997) allow the perceiver to achieve constancy. In the present study, however, constancy required only that the relation between the click and echo is encoded. One plausible strategy to achieve this is to directly encode the spectrum of the click and to “discount” its effect from the raw sensory response to the reflected sound—a strategy that is equivalent to models of constancy in other sensory processes (e.g., Epstein & Park, 1963; Mershon et al., 1981). This strategy would require an echolocator to be able to accurately encode the relevant properties of the click and echo separately. It is theoretically possible that participants could have solved our constancy task by making a judgment only on whether the two clicks sounded different or not (entirely ignoring the echo), but there are reasons why it is unlikely that participants adopted this strategy. First, in our study the click and echo were separated only by a very short temporal gap (<18 ms), and the two sounds (i.e., click and echo) themselves were very brief (<10 ms). Under such conditions the two sounds are very likely to be experienced as a single sound with a pitch that is inversely related to the duration of the gap (i.e., repetition pitch; Bilsen, 1966). Even if the click and echo were perceived as separate sound elements, the overall sound would have a “timbre” due to repetition pitch, making it impossible to process the click in isolation. Furthermore, constancy performance in the spectrum task was better for closer objects (and thus shorter time delays), whereas performance in the level task was better for more distant objects (and thus longer time delays). This strongly suggests that there might be separate strategies for achieving constancy across variation in level and spectrum, at least in the form of echolocation studied here, that is, where people listened to echolocation sounds via headphones. It is also worth noting that blind people have been found to be more accurate than sighted people in detecting a gap between two sounds (Muchnik et al., 1991), which might affect how different participants performed the task in our experiments. Yet, regardless of the exact process underlying performance, the finding that EEs performed better than SCs and BCs, while there was no difference between BCs and SCs in our experiments, highlight that group differences in performance that we observed must be due to experience with echolocation, rather than blindness per se.

It is also interesting to note that constancy performance in the spectrum task was better for closer objects, whereas performance in the level task was better for more distant objects. This not only suggests further functional independence between the two processes, but also gives an insight into the different underlying strategies. In the spectrum constancy task, because performance actually decreased with target distance, it is very unlikely that participants’ performance was driven by the perceptual separation of the click from the echo. In the level constancy task, however, it is possible that participants did attempt to perceptually separate the click from the echo, as their performance increased with target distance. Further study is needed, however, in order to identify the exact strategies used in these different constancy tasks.

It is somewhat surprising that EEs showed poor constancy across variations in the click’s level, given that EEs do vary the level of their click when actively echolocating according to task demands (Thaler et al., 2018, 2019). Importantly, this performance was not limited by their ability to discriminate the sound levels used in the constancy task—subjects were able to discriminate these individual components with high accuracy. It is also unlikely that this low performance was due to participants applying an incorrect strategy (e.g., attending to frequency rather than level). This is unlikely for three reasons. First, participants were told explicitly before each constancy task whether the differences would be carried by differences in level or spectrum. Second, participants completed the echo-acoustic training tasks prior to completing the constancy task, in which they made explicit judgments, with feedback, about the acoustic features that would be relevant for the constancy task. Third, in Experiment 5, when sighted participants completed the constancy task over three separate sessions, they received feedback on their accuracy after each trial but despite this, their performance did not improve on the level constancy task. High constancy ability in EEs might nonetheless be observed, however, if they were allowed to actively echolocate. In active echolocation, EEs would have access to motor feedback cues relating to mouth click production, and this might allow them to anticipate the sensory consequences of the mouth click (e.g., Baess et al., 2009; Cao et al., 2017; Martikainen et al., 2005; Schäfer & Marcus, 1973). Additionally, echolocation performance can improve with the presence of additional reflectors or in a reverberant environment (Schenkman & Nilsson, 2010; Tonelli et al., 2016), and it has been suggested that reverberation is a useful cue for supporting constancy in the perception of the loudness of a sound source (Zahorik & Wightman, 2001). EEs might, therefore, still be able to achieve high constancy across variations in level but only in active echolocation or in a more reverberant environment.

It is important to address some possible limitations of the present study. First, our three participant groups were not matched for age, with the largest discrepancy being between SCs (*M* = 23.1 years, *SD* = 3.3) and BCs (*M* = 52.6 years, *SD* = 12.3), but also for individual Experiments 1, 2, and 4 (see relevant experimental sections for details). The underlying reason is that it is not easy to recruit people who are blind. Importantly, however, in all components of Experiments 1, 2, and 4, we found no evidence of any performance differences between these two groups. Further to this, the results of the echo-acoustic training tasks used in Experiments 1 and 4 established that there were no group-level differences in the fundamental sensory processes that would be relevant for the constancy tasks. It is therefore unlikely that age differences between our participants affected performance in our study. Second, it is also important to discuss whether our findings would generalize to conditions beyond those tested in the present study. As this was the first study to measure constancy in a novel sensory skill, we adopted a reductionist experimental design in order to test our hypotheses under only the most essential conditions—that is, constancy for a single reflecting object in an anechoic chamber. As previously discussed, however, it is possible that constancy in echolocation will improve with the presence of additional reflectors or in a reverberant environment (Schenkman & Nilsson, 2010; Tonelli et al., 2016; Zahorik & Wightman, 2001), and this might constitute a more ecologically valid measure of an echolocator’s constancy ability. The results of our study, however, show that constancy is possible under even the most essential conditions, and further experiments can now test whether this generalizes to other scenarios.

Our results have implications for our understanding of the neural representations that might underlie sensory-independent perceptions. Specifically, our results raise the possibility that preexisting neural processes underlying constancy might be repurposed to support a form of constancy that is acquired through a novel sensory skill. These neural processes might, therefore, be best understood in terms of the physical properties of objects that they represent—especially those that can be perceived through multiple modalities (e.g., size, material)—rather than being bound to any specific sensory modality. This is consistent with recent theoretical developments supporting the view that the sensory brain is best understood as being task specific rather than modality specific (Amedi et al., 2017). Relatedly, the results of this study also have implications for rehabilitation following sensory loss. Our results demonstrate that perception through the use of a novel sensory skill can become functionally equivalent to normal sensory perception by supporting constant perceptual representations and, importantly, this can emerge rapidly through short training sessions. Given that constant representations are thought to be necessary for perceptual learning (Garrigan & Kellman, 2008) and guiding actions (Hatfield, 2009), the benefits from sensory restoration might be maximized with an approach that includes specific training to form perceptual representations that are constant with respect to the physical world.

## Figures and Tables

**Table 1 tbl1:** Details of 3 EEs and 17 BCs Who Participated in Experiments 1, 2, and 4

Participant	Gender	Age	Degree of vision loss	Cause and onset of vision loss	Echolocation use	Experiment
EE1	M	50	Total blindness	Enucleation due to retinoblastoma at 13 months	Daily; since early childhood/no exact age remembered	1, 2, 4
EE2	M	35	Total blindness	Gradual sight loss since birth due to glaucoma	Daily; since 12 years old	1, 2, 4
EE3	M	24	Total blindness	Enucleation at Age 19 due to sudden loss of vision (exact cause unknown)	Daily; since 12 years old	1, 2, 4
BC1	M	32	Total blindness	Retinopathy prematurity. Some vision in right eye from birth; retinal detachment in right eye at Age 12	None	1, 2, 4
BC2	M	67	Residual bright light perception	Leber’s amaurosis; from birth	None	1, 2, 4
BC3	M	48	Total blindness in left eye; residual bright light perception in right eye	Severe childhood glaucoma; 3 months old	None	2
BC4	M	63	Central vision in right eye; residual bright light perception in both eyes.	Glaucoma; poor vision since birth with increasing severity, registered blind Age 50	None	1, 4
BC5	F	59	Total blindness in left eye; peripheral vision in right eye	Stichler’s syndrome; retinal sciasis; from birth with increasing severity	None	2, 4
BC6	M	53	Residual bright light perception	Retinitis pigmentosa; official diagnosis Age 10. Gradual sight loss from birth	Some experience; very little regular use	1, 2
BC7	M	69	Residual bright light perception; some shape perception	Retinal dystrophy (exact cause unknown); official diagnosis Age 6–7	None	4
BC8	F	64	Total blindness	Undeveloped iris; from birth	None	1, 2, 4
BC9	M	39	Residual bright light perception	Retinitis pigmentosa; from Age 7–8		1, 2
BC10	F	56	Total blindness in left eye; residual bright light perception and some shape perception in right eye	Coloboma; from birth	None	4
BC11	F	62	Residual bright light perception	Retinal development abnormality; from birth	None	1
BC12	M	70	Residual bright light perception	Unknown cause; from birth	None	4
BC13	F	36	Residual bright light perception	Unknown cause; from birth	None	2
BC14	M	45	Total blindness	Ocular albinism. Gradual sight loss from birth	Some experience; very little regular use	1, 2, 4
BC15	M	45	Total blindness	Blood clot damaging optic nerve; Age 15	None	1, 4
BC16	M	37	Tunnel vision in both eyes	Retinitis pigmentosa; gradual from birth; official diagnosis Age 13	None	2
BC17	M	58	Total blindness	Retinoblastoma; enucleation at 2 years	None	1
*Note.* Some participants took part in more than one experiment. EE = expert echolocator; BC = blind control; M = male; F = female.						

## Footnotes

1 Medium spectrum/level emission recordings were not used, because they are not used in the main constancy task.

2 This was computed using the average number of participants in each group (due to the limited sample size of *n* = 3 for EEs).

3 G*Power requires an estimate of the correlation among repeated measures for this computation. We used our data from Experiments 3 and 5 to directly estimate this. Specifically, we calculated the correlation in constancy performance between Sessions 1 and 2 in Experiment 3 (*r*_(8)_ = .61), and between Sessions 1 and 2 in Experiment 5 (*r*_(8)_ = .23). We chose to use .23 for the minimum effect size calculations for both Experiments 3 and 5 because it gives the most conservative (highest) minimum detectable effect size.

4 No individual pairwise comparison between sessions (with Bonferroni correction) was significant (between 1 and 2, *p* = .53; between 1 and 3, *p* = .06; between 2 and 3, *p* = .50).
